# Proteins need extra attention: improving the predictive power of protein language models on mutational datasets with hint tokens

**DOI:** 10.1093/nargab/lqaf128

**Published:** 2025-09-26

**Authors:** Xinning Li, Ryann M Perez, Sam Giannakoulias, E James Petersson

**Affiliations:** Department of Chemistry, University of Pennsylvania, Philadelphia, PA 19104, United States; Department of Chemistry, University of Pennsylvania, Philadelphia, PA 19104, United States; Division for Advanced Computation, Sentauri Inc, Woodbine, MD 21738, United States; Department of Chemistry, University of Pennsylvania, Philadelphia, PA 19104, United States

## Abstract

In this computational study, we address the challenge of predicting protein functions following mutations by fine-tuning protein language models (PLMs) using a novel tokenization strategy, hint token learning (HTL). To evaluate the effectiveness of HTL, we benchmarked this approach across four pretrained models with varying architectures and sizes on four diverse protein mutational datasets. Our results showed significant improvements in weighted F1 scores in most cases when HTL was applied. To understand how HTL enhances protein mutational predictions, we trained sparse autoencoders on embeddings derived from the fine-tuned PLMs. Analysis of the latent spaces revealed that the number of activated residues within functional protein domains increased by PLM training with HTL. These findings indicate that PLMs fine-tuned with HTL may capture more biologically relevant representations of proteins. Our study highlights the potential of HTL to advance protein function prediction and provides insights into how HTL enables PLMs to capture mutational impacts at the functional level. All data and code are available at: https://github.com/ejp-lab/EJPLab_Computational_Projects/tree/master/HintTokenLearning.

## Graphical Abstract

**Graphical Abstract:**
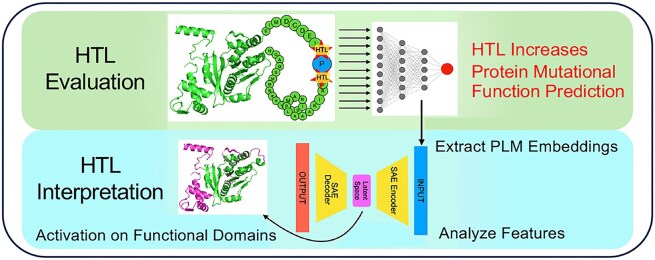

## Introduction

In recent years, large language models (LLMs) [1, 2] have reshaped the landscape of natural language processing (NLP) [3, 4], driving significant advances in tasks such as machine translation [5, 6], text generation [7], classification [8], summarization [9], and speech recognition [10]. The introduction of the transformer model by Vaswani *et al.* marked a significant milestone in LLM development [5]. The transformer architecture integrated insights from recurrent neural networks [11] and long short-term memory neural networks [12], while introducing a novel approach, the self-attention mechanism. Researchers have achieved remarkable capabilities using transformers in capturing long-range dependencies in sequential data, leading to state-of-the-art performance on many NLP tasks. An exemplary advancement in LLM that has captured the popular consciousness is the development of ChatGPT, a natural language model based on OpenAI’s GPT-3.5/4 architecture [7]. ChatGPT has garnered attention for its impressive ability to generate coherent and contextually relevant responses across diverse domains, and reached 100 million monthly active users just two months after its launch, making it the fastest-growing consumer application in history at that time [13].

Additionally, the success of the transformer architecture has extended beyond natural language to other domains, including biology. Protein sequences, which directly encode the complex 3-dimensional structures and functionalities of proteins, have become an intriguing target for LLM research. Building upon these breakthroughs, researchers have extended LLM techniques to the realm of proteins, leading to the development of protein language models (PLMs)—notably ProteinBERT [14], ProtBERT [15], ProtT5 [15], and ESM [16]. ProteinBERT adopts the BERT encoder-only architecture, using bidirectional self-attention to capture contextual relationships across an entire protein sequence. The ESM family also uses an encoder-only transformer but is distinguished by its large-scale pretraining on evolutionarily diverse UniRef50 sequences, enabling it to encode long-range residue couplings effectively. In contrast, ProtT5-XL is based on the T5 encoder–decoder architecture. Its span-corruption pretraining objective encourages the model to generate masked sequence patches, providing a generative perspective that complements the purely discriminative BERT-style objectives. By processing entire protein sequences simultaneously, these PLMs can uncover the subtle and complex relationships among amino acids, thereby enabling a range of predictive tasks. These span from token-level applications, such as predicting secondary structures, to sequence-level challenges, including the determination of subcellular localization [17].

Many studies have explored the potential of fine-tuning PLMs for various downstream tasks, such as protein engineering [18–20], protein function prediction [21] and annotation [22], and protein–protein [23, 24] and protein–ligand interactions [25]. Beyond prediction of specific outputs, PLMs have begun to build on the remarkable achievements of AlphaFold3 [26] in predicting protein three-dimensional structures. The trRosettaX-Single model [27] performs similarly to AlphaFold2 [28] without requirement of multiple sequence alignments (slow, CPU-based step to find most similar examples) greatly improving the speed of 3-dimensional structure prediction. Despite the widespread success of PLMs, relatively little attention has been given to their ability to accurately predict the functional impacts of protein mutations based solely on protein sequences. We hypothesize that this is because it is hard for models to capture incredibly small changes in the sequence (often only one out of 200 to 1000 residues) that can profoundly alter a protein’s structure or function [29]. To address this issue, we developed the hint token learning (HTL) strategy to emphasize such subtle changes, thereby enabling the model to better recognize and account for their impact on residues critical to protein function.

In this study, we proposed a novel machine learning (ML) strategy, which we call HTL, to enhance the ability of PLMs to discern and emphasize the critical impacts of mutations in protein sequences. Utilization of the HTL on fine-tuning pretrained models of different architectures and sizes across various downstream protein mutant functional prediction tasks demonstrates a noticeable improvement in predictive power. Beyond unveiling an approach for handling the complexities of mutational data, we also illustrate how HTL strengthens the capacity of PLMs to capture mutational impacts by training sparse autoencoders (SAEs) on embeddings derived from these fine-tuned models. Notably, incorporating HTL increases the number of activated residues in key protein functional domains, underscoring that HTL enhances predictive performance of PLMs by directing the models’ focus on biologically significant sites.

## Materials and methods

### Data preprocessing: RecA activity

The McGrew *et al.* [30] dataset was processed for ML by creating training, validation, and testing sets through multi-label stratification. Given that this set did not contain saturation mutagenesis data—i.e. comprehensive data where every possible amino acid substitution at each chosen position within the protein has been experimentally tested—and was limited to specific selected mutations for a single protein, we ensured that the number of mutations and the class label distribution were preserved across sets. Due to the smaller overall dataset size, we wanted to ensure a rigorous and challenging independent dataset for benchmarking. Therefore, we used multi-label stratification to produce an ∼72-13-15 training, validation, and testing set split for learning. The detailed data statistics are shown in Supplementary Table S1.

### Data preprocessing: PTEN and TPMT activity

The data for fine-tuning PLMs to predict PTEN and TPMT activities were derived from Matreyek *et al.* [31]. These datasets were partitioned into training, validation, and testing sets using an ∼80-10-10 split, with the balanced representation of both labels through multi-label stratification ensured. To further enhance out-of-group generalizability, we employed a residue position-based grouping method during splitting, such that no mutation positions were repeated across sets. This approach ensures that model training and evaluation rigorously test the model’s ability to generalize to novel positions. Detailed data statistics are provided in Supplementary Tables S2 and S3.

### Data preprocessing: KCNE1 activity

The data for training PLMs to predict the KCNE1 function were obtained from Muhammad *et al.* [32]. We split the dataset into training, validation, and test sets following an ∼80-10-10 ratio, ensuring balanced label distributions across each subset. Similarly to our procedures for the PTEN and TPMT datasets, we employed a position-based grouping approach. Under this method, all mutants occurring at the same sequence position were allocated to the same set, thereby compelling the model to generalize across all positions within the protein sequence. Detailed data statistics are presented in Supplementary Table S4.

### PLM training

We fine-tuned our classification models using pretrained PLMs available in Hugging Face, including ProtBERT (prot_bert) [15] and ProtT5 (prot_t5_xl_uniref50) [15] from Rostlab, as well as ESM2 models (esm2_t33_650M_UR50D and esm2_t48_15B_UR50D) [16] from Meta. For each case study, the final model was selected based on the best trial of Bayesian hyperparameter optimization conducted using the TPE sampler from the Optuna Python library [33]. A total of ten trials were performed, aiming to maximize the F1 score on the validation set. Each model was trained for up to 50 epochs, with early stopping applied if the validation score did not improve for 15 consecutive epochs. The fine-tuned models were then evaluated on a held-out test set to assess their predictive performance. Given the inherent class imbalance across our datasets, we reported weighted metrics rounded to two digits (weighted F1, weighted precision, and weighted recall) to mitigate imbalance effects.

### Hint tokenization

To address the challenge of accurately predicting protein functional changes from subtle sequence variations, we developed the novel tokenization strategy of HTL. Traditional tokenization strategies used in general protein language modeling typically tokenize amino acid sequences into individual amino acid residues without explicitly highlighting mutational positions. This standard approach can result in models struggling to detect subtle yet functionally significant mutations, as these changes often represent only minor perturbations within long sequences. In contrast, our HTL approach explicitly highlights mutation sites in the protein sequence by introducing two special tokens, [MUT_start] and [MUT_end], surrounding each mutated residue. HTL’s novelty lies in explicitly guiding the model’s focus toward critical mutation sites, an approach previously unexplored in protein language modeling for mutation prediction. Because this tokenization strategy provides hints of the mutation site to the models, we name it HTL.

The HTL strategy was implemented by directly marking mutation sites using special tokens. Specifically, sequences were modified to include [MUT_start] and [MUT_end] tokens flanking mutations relative to the wild-type construct. For instance, the mutation D26E (where the amino acid D at position 26 is mutated to E) was represented in the sequence as [MUT_start] E [MUT_end] at position 26. These hint tokens were inserted directly into the mutated sequence at the corresponding position. This modification was applied uniformly across all mutant samples.

To enable the models to recognize these new tokens, we modified the pretrained tokenizers from the Hugging Face Transformers library. Tokenizers were loaded using the from_pretrained method, and the [MUT_start] and [MUT_end] tokens were added to the tokenizer vocabulary using the add_tokens method. All mutated sequences were subsequently encoded using these updated tokenizers, ensuring that the hint tokens were properly recognized and incorporated into the input embeddings. In contrast, control sequences—used to benchmark the effectiveness of HTL—were tokenized using the standard method, without the hint tokens. In these cases, mutated residues were included directly in the sequence without any explicit indication of mutation, making them identical to wild-type inputs except for the substituted amino acid.

### HTL interpretation by SAE training and feature activation analysis

To better understand how HTL improves predictive accuracy, we aimed to provide deeper interpretability by analyzing the internal representations (embeddings) generated by our models. Specifically, we trained SAEs—unsupervised neural networks that learn simplified, compressed representations of data—on embeddings derived from the fine-tuned PLMs (ProtBERT, ESM-650M, and ProtT5).

Within the SAE latent space, specific amino acid residues become activated, meaning they significantly influence the encoded representations learned by the autoencoder. Such residue activation patterns offer an interpretable measure: activated residues are those that the model implicitly considers critical for predicting protein function. By comparing the quantity of activated residues before and after incorporating HTL, we aimed to reveal whether the model, guided explicitly by hint tokens, improved its ability to focus on biologically meaningful residues.

We evaluated the activated residue count within functional domains, regions of proteins that are experimentally annotated to perform specific biological activities or contribute directly to protein function (e.g. catalytic sites, binding sites, or regions involved in structural stability). Residues within these domains often hold critical importance, and enhanced activation in these areas after HTL application signals that the model’s improved performance by HTL is likely due to better recognition and prioritization of biologically significant regions.

To implement HTL interpretation discussed earlier, we followed the protocol of Simon *et al.* (2024) [34] and trained layer-wise SAEs on the embeddings produced by our fine-tuned ProtBERT, ESM-650M, and ProtT5 models. The encoded representations from the latent space were subsequently extracted for each layer to analyze protein sequences. To quantify the impact of the HTL strategy on residue activation patterns, we calculated the relative change in activated residue numbers using the following equations (Equations 1 and 2):


(1) \begin{eqnarray*}
{{A}_{{\rm tokenization\_strategy}}} = \frac{1}{{{{N}_l}}}\mathop \sum \limits_{j = 1}^{{{N}_l}} \frac{1}{{{{N}_s}}}\mathop \sum \limits_{i = 1}^{{{N}_s}} \frac{{{{A}_{ij}}}}{R}.
\end{eqnarray*}

Equation (1) defines ${{A}_{{\rm tokenization\_strategy}}}$, which represents the average percentage of the number of activated residues within functional domains relative to the total number of residues in these domains across test samples and model layers. ${{N}_l}$ denotes total number of model layers, ${{N}_s}$ represents the total number of test samples and ${{A}_{i,j}}$ is the number of activated residues in functional domains for sample *i*-th in layer *j*-th. The term *R* refers to the total number of residues in functional domains, which remains constant for a given protein because the number of residues in functional domains does not vary within the calculations for the same protein.


(2) \begin{eqnarray*}
{\mathrm{\Delta }}{{A}_{{\rm {\rm rel}}}} = \ \frac{{{{A}_{HTL}} - \ {{A}_{NO\_HTL}}}}{{Max\left( {{{A}_{HTL}},\ {{A}_{NO\_HTL}}} \right)}}{\mathrm{\ }}.
\end{eqnarray*}

Equation (2) is used to calculate ${\mathrm{\Delta }}{{A}_{{\rm rel}}}$, which is the relative change in ${{A}_{{\rm tokenization\_strategy}}}$ after applying the HTL strategy. The teams ${\mathrm{\Delta }}{{A}_{HTL}}$ and ${\mathrm{\Delta }}{{A}_{NO\_HTL}}$ are derived from Equation (1), and represent the average percentages of the number of activated residues within functional domains relative to the total number of residues in these domains across test samples and model layers —with and without the HTL strategy, respectively.

## Results

### HTL evaluation

#### ProtBERT fine-tuning with HTL boosts RecA activity prediction

The RecA protein is an attractive drug target due to its critical role in forming nucleoprotein filaments, a process essential for conferring antibiotic resistance [35]. Identifying key residues that influence RecA activity and designing inhibitors to disrupt its function are therefore important objectives. However, experimentally measuring bioactivity changes for large numbers of combinatorial mutations remains challenging, limiting our understanding of the molecular mechanisms underpinning RecA function. To address this challenge, we explored the potential of training PLMs to rapidly and cost-effectively predict RecA bioactivity from the primary sequences of its mutants.

We fine-tuned the ProtBERT model (a lighter-weight foundational PLM accessible to the hardware of most computational researchers) on a RecA bioactivity dataset compiled from McGrew *et al.* [30]. This dataset comprises nearly 1000 RecA mutants, each labeled according to its activity relative to the wild-type protein. These labels were used to make a three-part classifier with activities greater than or equal to wild-type (rec+), similar, but less than wild-type (rec±), and no activity (rec−).

**Figure 1.**
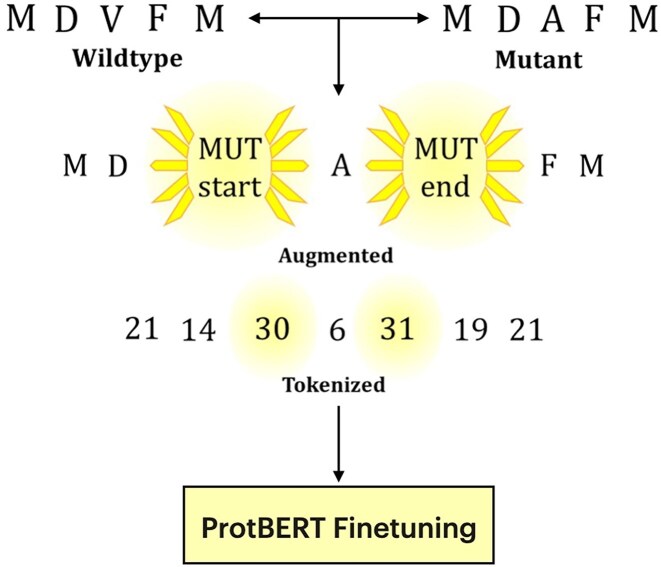
Diagram of the HTL process illustrated on a single-point mutant. A fragment of the wild-type sequence (MDVFM) has a V → A mutation at position 3 (MDAFM). Augmentation step: The mutated residue is flanked by the hint tokens [MUT_start] and [MUT_end], making the change explicit in the input string. Tokenization step: Every amino acid as well as the two new hint tokens are converted to their token IDs by the modified tokenizer (e.g. M → 21, [MUT_start] → 30, and [MUT_end] → 31). Finally, the resulting token ID sequence is passed to the downstream PLM (here, ProtBERT) for fine-tuning.

We observed an extremely poor ability to classify the RecA dataset even after hypertuning (weighted F1: 0.41). Our overarching goal is to leverage PLMs for high-throughput identification of residues critical to RecA function. At this point, rather than investigate alternative approaches such as full-atom simulation-based strategies that are cost prohibitive, we decided to devise a new language model learning strategy, which could augment the quality of PLMs in the protein mutant task. Our strategy was to train the PLM on the same sequences, which have been tokenized with the two new special tokens (MUT_start and MUT_end) surrounding the mutation site(s) to help the model focus on these changes (HTL; Fig. 1). After applying HTL, we observed a significant improvement in the ability of ProtBERT to classify the RecA dataset (weighted F1: 0.58) with a nearly 17% weighted F1 increase (Supplementary Table S5).

#### ProtBERT fine-tuning with HTL also enhances PTEN, TPMT, and KCNE1 activity prediction

**Figure 2.**
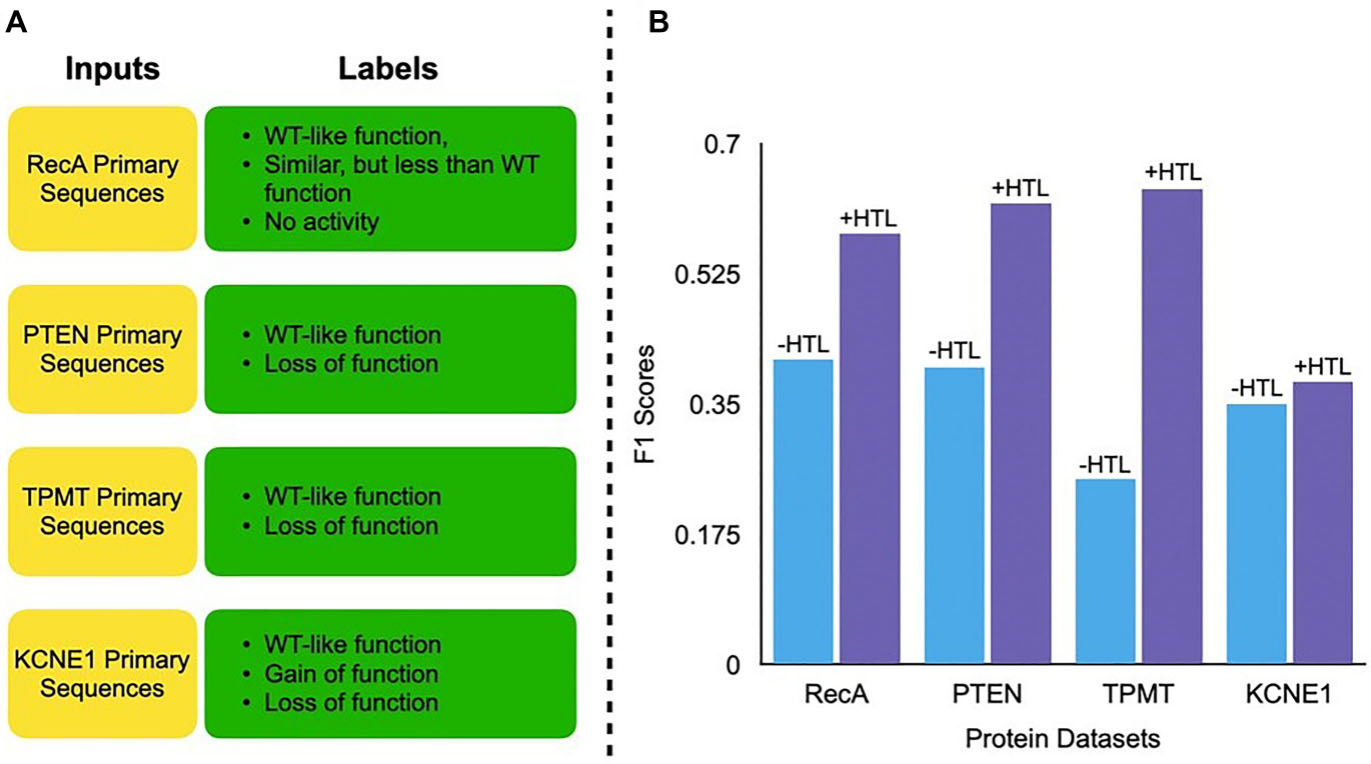
Assessment of the effectiveness of the HTL strategy during ProtBERT fine-tuning. (**A**) Dataset overview. Each yellow block lists the input provided to the model (primary amino acid sequence of a protein mutant), and the adjacent green block lists the discrete functional labels used for supervised learning. (**B**) Performance Metrics. Weighted F1 of ProtBERT fine-tuning on multiple protein mutational datasets with and without HTL. Blue bars indicate weighted F1 scores from models trained without HTL, and purple bars indicate weighted F1 scores from models trained with HTL.

To assess whether the HTL strategy improves protein function predictions beyond RecA, we assembled three additional mutational datasets—PTEN [31], TPMT [31], and KCNE1 [32]—each derived from deep mutational scanning experiments. In these experiments, many protein variants were evaluated for fitness, and activity labels were assigned using carefully defined fitness score thresholds. As illustrated in Fig. 2A, the input to each model is a protein primary sequence containing one or more point mutations relative to the wild-type construct. No structural, evolutionary, or experimental features are used. The output is a discrete label that reflects the predicted functional consequence of that variant, based on activity measurements from high-throughput mutational scanning. For instance, PTEN and TPMT samples are labeled as wild-type-like or loss of function, while KCNE1 variants are classified into gain of function, wild-type-like, or loss of function. These labels reflect experimentally defined fitness bins and serve as the classification targets. Each variant is treated independently, and our models are trained to learn mapping from raw sequence (with or without hint tokens) to one of the predefined functional categories. These new datasets, ranging from ∼2000 to over 4000 variants, are substantially larger than our original RecA dataset that has around 1000 variants (Supplementary Tables S1–S4). By incorporating datasets of different scales and complexities, we aimed to more thoroughly evaluate the robustness and scalability of the HTL approach.

Figure 2B shows the weighted F1 score of ProtBERT fine-tuning on four protein mutational datasets, comparing results obtained with and without the HTL approach. Each dataset is class-imbalanced and two of these datasets involve more than two activity categories (Supplementary Tables S1–S4 and Fig. 2A). We therefore adopt weighted F1 as our primary metric for the following reasons: (i) Multi-class suitability: by averaging precision and recall in proportion to class prevalence, weighted F1 gives a single, prevalence-aware score that provides easier interpretability and works equally well for binary and multi-class label sets—something that other common metrics like receiver operating characteristic area under the curve (ROC-AUC), precision-recall area under the curve (PR-AUC), and Matthews correlation coefficient (MCC) do not natively provide. (ii) Scientific cost of errors: in academic applications, both false positives (mis-flagging neutral variants) and false negatives (missing deleterious variants) carry high experimental cost, and weighted F1 captures this balance transparently.

These cross-domain findings indicate that HTL substantially boosts weighted F1 scores on independent test sets, outperforming standard fine-tuning methods without introducing the complexity often seen in structure-based [36, 37] or alignment-based approaches [38]. In most cases, the HTL strategy elevates performance significantly from the baseline models trained without the HTL. For instance, on the TPMT dataset, the model’s weighted F1 score increases from ∼0.25 without HTL to ∼0.64 with HTL—a remarkable 156% enhancement (Supplementary Table S7). Notably, when fine-tuned on PTEN without HTL, ProtBERT failed to learn meaningful distinctions and simply defaulted to predicting the majority class (Supplementary Table S9 and S10). The KCNE1 dataset, however, presents an interesting outlier. Although the HTL strategy still improves performance, the gain is more modest (∼9%) compared to the substantial advances seen in the other datasets. KCNE1 functions as an auxiliary subunit (minK) regulating the voltage-gated potassium channel KCNQ1 [39]. Its regulatory role, rather than a single enzymatic function, can lead to more nuanced sequence-to-function relationships. Small sequence changes in KCNE1 might have large downstream impacts only under specific conditions, making it harder for a model trained solely on sequence data to learn clear predictive patterns.

### Investigation of HTL with other pretrained models

To further assess the generalizability of the HTL strategy, we evaluated its impact on PLMs fine-tuned from diverse pretrained models of varying architectures and sizes, including ESM-650M, ProtT5-XL-UniRef50, and ESM-15B. We compared the performance of these models with and without the HTL strategy, focusing particularly on the weighted F1 score to address data imbalance (Supplementary Tables S5–S8 and Table 1). In most cases, HTL enhances model performance, enabling ProtT5 with HTL to achieve the highest weighted F1 scores on the RecA, PTEN, and KCNE1 datasets. For instance, on the RecA dataset, ESM-650M with HTL demonstrated a remarkable 60% improvement in weighted F1 score compared to the model trained without HTL. Similarly, ESM-15B with HTL consistently outperformed across multiple datasets, including RecA, PTEN, and TPMT. These results highlight the versatility and effectiveness of HTL in enhancing the predictive accuracy of models, regardless of the size or architecture of the pretrained model, across diverse protein mutational tasks.

**Table 1.** Performance metrics of ProtBERT, ESM-650M, ProtT5-XL-UniRef50, and ESM-15B fine-tuning on multiple protein mutational datasets with and without HTL

Weighted F1	ProtBERT(450M)		ESM(650M)		ProtT5(3B)		ESM(15B)	
	NO_HTL	HTL	NO_HTL	HTL	NO_HTL	HTL	NO_HTL	HTL
RecA	0.41	**0.58**	0.35	**0.56**	0.67	0.71*	0.57	**0.59**
PTEN	0.4	**0.63**	0.5	**0.57**	0.69	0.71*	0.4	**0.62**
TPMT	0.25	**0.64**	0.67	**0.7**	0.71*	0.65	0.24	**0.51**
KCNE1	0.35	**0.38**	**0.55**	0.46	0.41	0.6*	**0.49**	0.46

Models are compared before and after applying the HTL strategy. Bolded values indicate the highest weighted F1 score among models trained on the same dataset and initialized from the same pretrained model. Values marked with an asterisk (*) represent the highest weighted F1 score achieved by any model on each dataset.

Although ProtT5 without HTL performs best on TPMT, the ESM-650M model with HTL achieves a nearly identical weighted F1 score, trailing by only 0.01. This result is particularly noteworthy since ESM-650M is substantially smaller and presumably less information-rich due to its reduced pretraining complexity. The fact that HTL narrows this performance gap underscores its effectiveness in improving predictive accuracy for protein mutational effects. By enabling smaller models to achieve comparable results, the HTL strategy reduces the need for large-scale architectures, ultimately saving training time and increasing overall efficiency.

As previously discussed, KCNE1 represents a notably challenging predictive task. Most models yield bad results on this dataset, underscoring its inherent complexity. However, ProtT5 coupled with HTL achieves a weighted F1 score of ∼0.6—an improvement of around 46%. This substantial gain reinforces HTL’s utility, even for difficult scenarios where conventional fine-tuning methods struggle to move beyond baseline performance.

Although HTL did not always provide substantial improvements in every tested situation, we never observed any drastic degradation in performance when using HTL either. HTL fairly consistently enhanced the ability of PLMs to predict mutational effects across multiple pretrained models of varying sizes and architectures on diverse protein datasets. These results highlight HTL as a promising and broadly applicable strategy for improving predictive accuracy in protein mutational tasks, while also reducing the reliance on extensive model sizes.

### HTL interpretation

**Figure 3.**
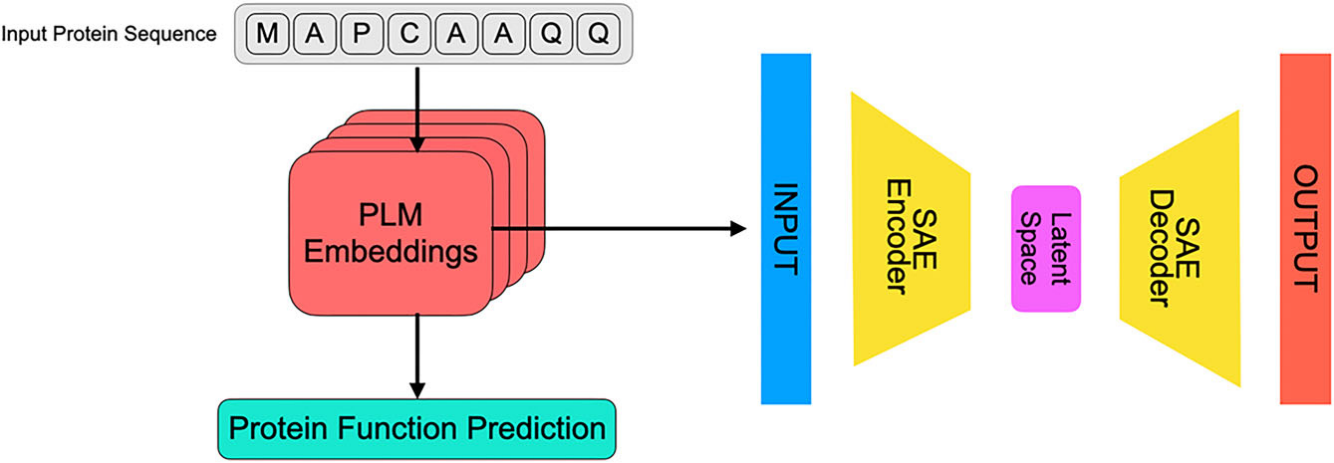
Scheme for training SAEs on the embeddings of PLMs.

Building on the approach of Simon *et al.* (2024) [34], we trained SAEs on embeddings derived from the fine-tuned ProtBERT, ESM_650M, and ProtT5 models (Fig. 3). These models were chosen over ESM_15B due to their stronger performance and greater training efficiency in predicting protein functional changes upon mutation. Within the SAE framework, the activation of specific amino acid residues in the latent space likely indicates their importance for the model’s predictions. By comparing residue activation patterns before and after applying the HTL strategy, we sought to elucidate how HTL enhances the predictive power of these models.

**Figure 4.**
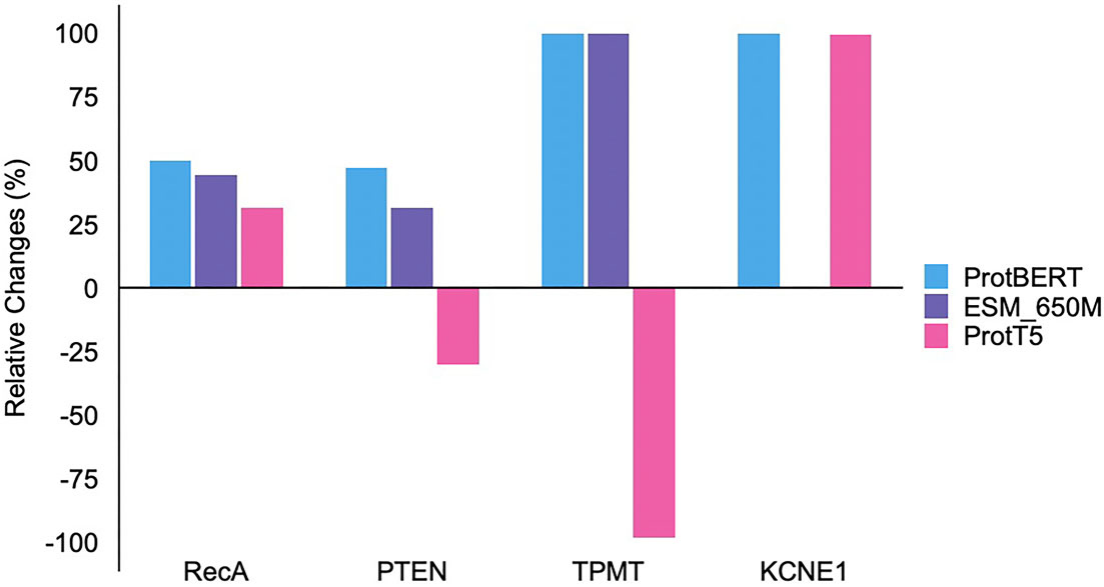
Relative changes of the number of activated residues in functional domains after applying the HTL strategy across all models and datasets in deeper layers of models. The blue, purple, and pink bars represent the relative changes of ProtBERT, ESM-650M, and ProtT5, respectively.

Supplementary Table S11 reports the relative increase in activated, functionally relevant residues after incorporating HTL, averaged across all layers. The results show that SAEs trained on HTL-refined embeddings generally activate more residues located within known functional domains (except for models where performance changes were minimal or slightly declined). This suggests that HTL encourages embeddings to highlight more critical, function-driving residues, thus enabling PLMs to better identify and interpret regions essential for protein function. We also examined these activation patterns in deeper embedding layers. Deeper layers of PLMs are believed to encode more complex, contextually rich information that often corresponds to higher-level biological concepts, such as functional domains or allosteric sites. By analyzing changes in these deeper layers, we can further validate HTL’s ability to enhance the capture of more biologically meaningful features by the models. Our findings confirm that the trend of increased residue activation within functional domains following HTL persisted in deeper layers for most cases (Fig. 4), except for a few instances where model performance changes after applying HTL were minimal or slightly declined (ProtT5_PTEN, ProtT5_TPMT, and ESM_650M_KCNE1). Nevertheless, these findings collectively indicate that HTL enriches the biologically relevant information captured by models, thereby better equipping PLMs to predict how mutations influence protein function.

## Discussion

Our findings demonstrate that the HTL strategy substantially improves the ability of PLMs to predict the functional impact of mutations directly from primary sequences. By applying HTL, we consistently observed marked increases in weighted F1 scores across multiple protein mutational datasets—RecA, PTEN, TPMT, and KCNE1—spanning various scales and complexities. The noteworthy gains were observed in challenging classification tasks that previously approached near-random performance without HTL. For example, ProtBERT fine-tuning coupled with HTL enhanced TPMT activity prediction from a weighted F1 score of 0.25 to 0.64, elevating performance by as much as 156%. Similar patterns emerged for RecA and PTEN; the application of HTL significantly boosts the predictive power of PLMs.

Still, certain datasets such as KCNE1 remain challenging. Despite notable improvements with HTL, this dataset’s predictive task remains exceptionally difficult, as underscored by historically modest performance across numerous modeling approaches (Notin *et al.* 2023) [40]. The limited gains for KCNE1 even after HTL highlight the inherent challenges of deriving biologically relevant features solely from primary sequences, particularly for proteins with subtle, context-dependent functional determinants that may require additional structural or evolutionary insights. Nevertheless, the HTL approach helped ProtT5 fine-tuned on KCNE1 achieve a statistically significant weighted F1 score of 0.6—an approximate 46% increase—demonstrating its ability to yield meaningful improvements even for particularly intractable problems.

Beyond improving predictive power, HTL offers a versatile and easily integrated approach that does not require the extensive computational overhead or domain-specific information demanded by structure- or alignment-based methods. Notably, the improvements conferred by HTL were not limited to a single underlying model. We observed positive impacts across diverse architectures and sizes, including ProtBERT, ESM-650M, ProtT5-XL-UniRef50, and ESM-15B. The consistent benefits observed, especially for smaller and less complex PLMs, point toward HTL’s generalizability and scalability. By enabling smaller models to perform on par with larger, more complex ones, HTL boosts training efficiency and alleviates hardware constraints. These findings encourage the broader adoption of HTL, which can help democratize advanced protein function prediction by reducing reliance on massive models and the associated computational burden.

Importantly, our exploration of model embeddings via SAEs further illustrates the mechanistic underpinnings of HTL’s success. The introduction of hint tokens appears to direct a model’s latent representations more toward functionally critical residues and domains. By explicitly flagging mutation sites, the model’s attention is guided toward the sequence features most relevant for functional determination that could possibly be influenced by mutations. Post-HTL embeddings more strongly activated known functional residues, and these enhancements persisted into deeper model layers, suggesting that HTL helps models encode richer, context-dependent biological information. Although this trend did not hold in every instance—particularly when performance changes were minimal or slightly declined—these findings still provide valuable insight into how sequence-based models can be coaxed into learning more interpretable and biologically meaningful features.

Taken together, these results highlight the promise of HTL as a broadly applicable, computationally accessible strategy to improve sequence-based protein function prediction. By directing PLMs to focus on biologically significant sites, HTL enhances their ability to predict the functional consequences of mutations without incurring substantial computational costs. This approach may facilitate protein engineering by swiftly flagging critical residues for experimental validation, thereby saving significant time and resources. Future work could involve integrating HTL with complementary approaches, such as residue-level evolutionary couplings [41] or structural modeling [42]. Evolutionary couplings highlight pairs of residues that tend to mutate together across species, signaling that they function in concert. Flagging these co-evolving positions would draw the model’s attention to functionally linked sites. Likewise, a 3D structure reveals residues that are physically adjacent even when they are distant in the sequence; marking these spatial neighbors would cue the model to critical contacts. Layering such alignment- and structure-derived hints on top of HTL could steer the model toward the residues that truly govern activity, leading to more accurate and interpretable predictions. In our own laboratory, we anticipate using HTL to study the effects of mutations on the amyloid aggregation of proteins such as α-synuclein, designing aminoacyl tRNA synthetase enzymes for the incorporation of unnatural amino acids as well as identifying nonperturbing sites for their incorporation in proteins of interest, and utilizing our recent cryo-electron microscopy structure of the functional complex of RecA and LexA in combination with RecA HTL models described to understand the effects of mutations on antibiotic resistance [43–47].

## Supplementary Material

lqaf128_Supplemental_File

## Data Availability

The data used to train our models, including all training, validation, and testing splits are available on our GitHub at https://github.com/ejp-lab/EJPLab_Computational_Projects/tree/master/HintTokenLearning/Data. The code for HTL and a guide to reproducing the results demonstrated herein is available on our GitHub at https://github.com/ejp-lab/EJPLab_Computational_Projects/tree/master/HintTokenLearning/Model_Training. The data are also available on Figshare at https://figshare.com/articles/software/Hint_Token_Learning/28443833?file=52469846 and https://doi.org/10.6084/m9.figshare.28443833.v3.
