# Supplementary material for: Proteins need extra attention: improving the predictive power of protein language models on mutational datasets with hint tokens
Source: NAR Genom Bioinform. 2025 Sep 26;7(3):lqaf128. doi: 10.1093/nargab/lqaf128 (PMC12464817; doi:10.1093/nargab/lqaf128)
Supplement: lqaf128_Supplemental_File [file lqaf128_supplemental_file.pdf]

Electronic Supplementary Information

Proteins Need Extra Attention: Improving the Predictive Power of Protein Language Models on Mutational Datasets with Hint Tokens

Xinning Li,[a] Ryann Perez,[a] Sam Giannakoulias,*[a,b] and E. James Petersson*[a]

[a]Department of Chemistry, University of Pennsylvania, Philadelphia, Pennsylvania 19104, USA

[b]Division for advanced computation, Sentauri Inc, Woodbine, Maryland 21738, USA

* To whom correspondence should be addressed. S.G.: Tel: 1-4439098268; Email: samgiannakoulias@sentauriai.com; E.J.P.: Tel: 1-215746221; Email: ejpetersson@sas.upenn.edu

Table of Contents:

1. **Software.. S2**
2. **Datasets... S2**
3. **Model Training S4**
4. **Model Embedding Analysis S10**

Software

The software used for this work can be found in the conda environment yml file alongside installation instructions at the following link: https://github.com/ejp-lab/EJPLab_Computational_Projects/blob/master/HintTokenLearning/environment.yml

Dataset

Table S1, S2, S3, and S4 show the statistics of RecA dataset, PTEN dataset, TPMT dataset, and KCNE1 dataset. All machine learning datasets were created as described in the main text. For reproducibility, these datasets can be found at our GitHub through the following link:

https://github.com/ejp-lab/EJPLab_Computational_Projects/tree/master/HintTokenLearning/Data

Table S1. RecA dataset statistics

RecA	Training (~72%)	Validation (~13%)	Testing (~15%)
WT-like function	157	28	30
Similar, but less than WT function	181	32	38
No activity	353	62	71
Total	691	122	139

Table S2. PTEN dataset statistics

PTEN	Training (~80%)	Validation (~10%)	Testing (~10%)
WT-like function	1,866	247	228
Loss of function	1,428	160	183
Total	3,294	407	411

Table S3. TPMT dataset statistics

TPMT	Training (~80%)	Validation (~10%)	Testing (~10%)
WT-like Function	1,772	240	256
Loss of function	1,164	122	135
Total	2,936	362	391

Table S4. KCNE1 dataset statistics

KCNE1	Training (~80%)	Validation (~10%)	Testing (~10%)
Loss of function	794	111	98
WT-like function	469	32	75
Gain of function	592	81	65
Total	1,855	224	238

Model Training

Hyperparameter Tuning

Our models were hyperparameter-tuned by Optuna Bayesian optimization and the best parameters for all the models can be found at our GitHub through the following link: https://github.com/ejp-lab/EJPLab_Computational_Projects/tree/master/HintTokenLearning/Best_Model_Params

Comprehensive testing metrics for RecA, PTEN, TPMT, and KCNE1

Table S5-8 are comprehensive testing metrics for RecA dataset, PTEN dataset, TPMT dataset and KCNE1 dataset.

Table S5. Comprehensive testing metrics of RecA dataset.

	Pretrained Model	Finetuning Method	F1	ACC	PPV	TPR	MCC
ProtBERT_RecA_NO_HTL	ProtBERT	Traditional	0.41	0.39	0.47	0.39	0.061
ProtBERT_RecA_HTL		HTL	0.58	0.57	0.61	0.57	0.34
ESM_650M_RecA_NO_HTL	ESM_650M	Traditional	0.35	0.38	0.62	0.38	0.22
ESM_650M_RecA_HTL		HTL	0.56	0.6	0.57	0.6	0.32
ProtT5_RecA_NO_HTL	ProtT5	Traditional	0.67	0.66	0.67	0.66	0.46
ProtT5_RecA_HTL		HTL	0.71	0.71	0.71	0.71	0.53
ESM_15B_RecA_NO_HTL	ESM_15B	Traditional	0.57	0.55	0.71	0.55	0.39
ESM_15B_RecA_HTL		HTL	0.59	0.58	0.67	0.58	0.39

F1: weighted F1; ACC: Accuracy; PPV: weighted Positive Predictive Value (weighted Precision); TPR: weighted True Positive Rate (weighted Recall); MCC: Matthews Correlation Coefficient

Table S6. Comprehensive testing metrics of PTEN dataset.

	Pretrained Model	Finetuning Method	F1	ACC	PPV	TPR	MCC
ProtBERT_RecA_NO_HTL	ProtBERT	Traditional	0.4	0.55	0.31	0.55	0.00
ProtBERT_RecA_HTL		HTL	0.62	0.62	0.62	0.62	0.22
ESM_650M_RecA_NO_HTL	ESM_650M	Traditional	0.5	0.61	0.75	0.61	0.25
ESM_650M_RecA_HTL		HTL	0.57	0.58	0.58	0.58	0.14
ProtT5_RecA_NO_HTL	ProtT5	Traditional	0.69	0.72	0.75	0.72	0.44
ProtT5_RecA_HTL		HTL	0.71	0.72	0.73	0.72	0.44
ESM_15B_RecA_NO_HTL	ESM_15B	Traditional	0.4	0.55	0.31	0.55	0.00
ESM_15B_RecA_HTL		HTL	0.62	0.62	0.62	0.62	0.22

F1: weighted F1; ACC: Accuracy; PPV: weighted Positive Predictive Value (weighted Precision); TPR: weighted True Positive Rate (weighted Recall); MCC: Matthews Correlation Coefficient

Table S7. Comprehensive testing metrics of TPMT dataset

	Pretrained Model	Finetuning Method	F1	ACC	PPV	TPR	MCC
ProtBERT_RecA_NO_HTL	ProtBERT	Traditional	0.25	0.38	0.7	0.38	0.096
ProtBERT_RecA_HTL		HTL	0.64	0.64	0.67	0.64	0.26
ESM_650M_RecA_NO_HTL	ESM_650M	Traditional	0.67	0.66	0.69	0.66	0.31
ESM_650M_RecA_HTL		HTL	0.7	0.69	0.7	0.69	0.34
ProtT5_RecA_NO_HTL	ProtT5	Traditional	0.71	0.73	0.72	0.73	0.37
ProtT5_RecA_HTL		HTL	0.65	0.64	0.67	0.64	0.27
ESM_15B_RecA_NO_HTL	ESM_15B	Traditional	0.24	0.37	0.65	0.37	0.069
ESM_15B_RecA_HTL		HTL	0.51	0.52	0.7	0.52	0.25

F1: weighted F1; ACC: Accuracy; PPV: weighted Positive Predictive Value (weighted Precision); TPR: weighted True Positive Rate (weighted Recall); MCC: Matthews Correlation Coefficient

Table S8. Comprehensive testing metrics of KCNE1 dataset.

	Pretrained Model	Finetuning Method	F1	ACC	PPV	TPR	MCC
ProtBERT_RecA_NO_HTL	ProtBERT	Traditional	0.35	0.39	0.59	0.39	0.14
ProtBERT_RecA_HTL		HTL	0.38	0.42	0.61	0.42	0.19
ESM_650M_RecA_NO_HTL	ESM_650M	Traditional	0.55	0.55	0.56	0.55	0.31
ESM_650M_RecA_HTL		HTL	0.46	0.46	0.47	0.46	0.19
ProtT5_RecA_NO_HTL	ProtT5	Traditional	0.41	0.43	0.6	0.43	0.20
ProtT5_RecA_HTL		HTL	0.6	0.61	0.6	0.61	0.40
ESM_15B_RecA_NO_HTL	ESM_15B	Traditional	0.49	0.5	0.59	0.5	0.29
ESM_15B_RecA_HTL		HTL	0.46	0.47	0.49	0.47	0.22

F1: weighted F1; ACC: Accuracy; PPV: weighted Positive Predictive Value (weighted Precision); TPR: weighted True Positive Rate (weighted Recall); MCC: Matthews Correlation Coefficient

Table S9. The classification report of ProtBERT trained on PTEN with HTL

	Precision	Recall	F1 score	Support
Loss of Function	0.58	0.54	0.56	183
WT-like function	0.65	0.68	0.67	228
Accuracy			0.62	441
Macro average	0.61	0.61	0.61	441
Weighted average	0.62	0.62	0.62	441

Table S10. The classification report of ProtBERT trained on PTEN without HTL

	Precision	Recall	F1 score	Support
Loss of Function	0.00	0.00	0.00	183
WT-like function	0.55	1.00	0.71	228
Accuracy			0.55	411
Macro average	0.28	0.50	0.36	411
Weighted average	0.38	0.55	0.40	411

Model Embedding Analysis

We trained sparse autoencoders (SAEs) on embeddings derived from the fine-tuned ProtBERT, ESM_650M, and ProtT5 models for each layer. Subsequentially, we extracted encoded representations from the latent space and calculated the average change in the number of activated residues within functional domains across all layers using Equation (1) in the main text. We observed an increase in the number of activated residues within functional domains following the application of HTL, with few exceptions where model performance showed minimal changes or slight declines. Despite these exceptions, the findings collectively suggest that HTL enhances the capture of biologically relevant information, thereby improving the ability of PLMs to predict the impact of mutations on protein function.

Table S11. Relative changes of the number of activated residues in functional domains by SAE after applying the HTL strategy across all layers

All	ProtBERT	ESM-650M	ProtT5
RecA	18.2%	67.1%	99.8%
PTEN	26.7%	21.1%	0%
TPMT	95.8%	-23.5%	-5.6%
KCNE1	71.4%	45.9%	54.8%
